# Aortic Valve Intervention in Patients with Aortic Stenosis and Small Annulus

**DOI:** 10.31083/RCM26738

**Published:** 2025-03-24

**Authors:** Ahmed Abdalwahab, Muntaser Omari, Mohammad Alkhalil

**Affiliations:** ^1^Cardiothoracic Centre, Freeman Hospital, NE7 7DN Newcastle Upon Tyne, UK; ^2^Cardiovascular Medicine Department, Faculty of Medicine, Tanta University, 31527 Gharbia Governorate, Egypt; ^3^Translational and Clinical Research Institute, Newcastle University, NE1 7RU Newcastle Upon Tyne, UK

**Keywords:** transcatheter aortic valve implantation, patient prosthetic mismatch, effective orifice area, computed tomography, surgical aortic valve replacement, body surface area

## Abstract

Over the last two decades, the management of aortic stenosis has undergone significant transformation due to developments in surgical techniques and the introduction of transcatheter aortic valve implantation (TAVI). These transformations have enabled improved patient selection and treatments to be tailored based on individual clinical and anatomical characteristics. Both surgical and transcatheter options have resulted in reduced mortality and enhanced quality of life for patients with aortic stenosis. Nonetheless, treating patients with small aortic annulus remains challenging despite advances in current technology. The insertion of a small prosthetic valve, leading to patient prosthetic mismatch, has been associated with heart failure hospitalization, early structural valve degeneration, and long-term mortality. Although aortic root enlargement was historically employed to address this issue, stentless and sutureless valves in the supra-annular position and, more recently, TAVI have emerged as alternative treatments for patients with small annulus and severe aortic stenosis. This review will provide an overview of the prevalence and anatomical characteristics of patients with aortic stenosis and small annulus. Additionally, we will discuss current treatment options, including surgery and TAVI, used to mitigate procedural and long-term adverse outcomes in this group.

## 1. Introduction

The management of aortic stenosis has transformed over the last two decades. Advances in surgical techniques and the introduction of transcatheter aortic valve implantation (TAVI) have allowed better patient selection and tailored treatment according to individual patient characteristics [1, 2]. Despite its poor prognosis when left untreated, aortic stenosis risk can be significantly modified using surgical or transcatheter options, resulting in reduced mortality and improved quality of life for patients [3, 4].

Nonetheless, certain anatomical subsets remain challenging when treating patients with severe aortic stenosis. The presence of a small annulus was previously linked to procedural morbidity and mortality. However, the hemodynamic consequences of inserting a small prosthetic valve, promoting patient prosthetic mismatch (PPM), were recognized early [5, 6]; this phenomenon was linked to early structural valve degeneration and long-term mortality [7]. Historically, aortic root enlargement has been applied to address this issue. Subsequently, stentless and sutureless valves in supra-annular positions were proposed to overcome the inferior hemodynamic results of stented surgical valves. More recently, TAVI has emerged as an alternative option to treat patients with severe aortic stenosis, particularly those with small annulus. Compared to surgical aortic valve replacement, the lower gradients and larger effective orifice area (EOA) associated with TAVI would suggest that TAVI may be the optimal treatment for patients with severe aortic stenosis and small annulus [8, 9, 10].

This review will provide an overview of the prevalence and characteristics of patients with aortic stenosis and small annulus. Moreover, we will discuss the current treatment options, including surgical and transcatheter strategies, to mitigate procedural and long-term adverse outcomes.

## 2. Demographics and Characteristics of Patients with Small Aortic Annulus

**Fig. 1.**
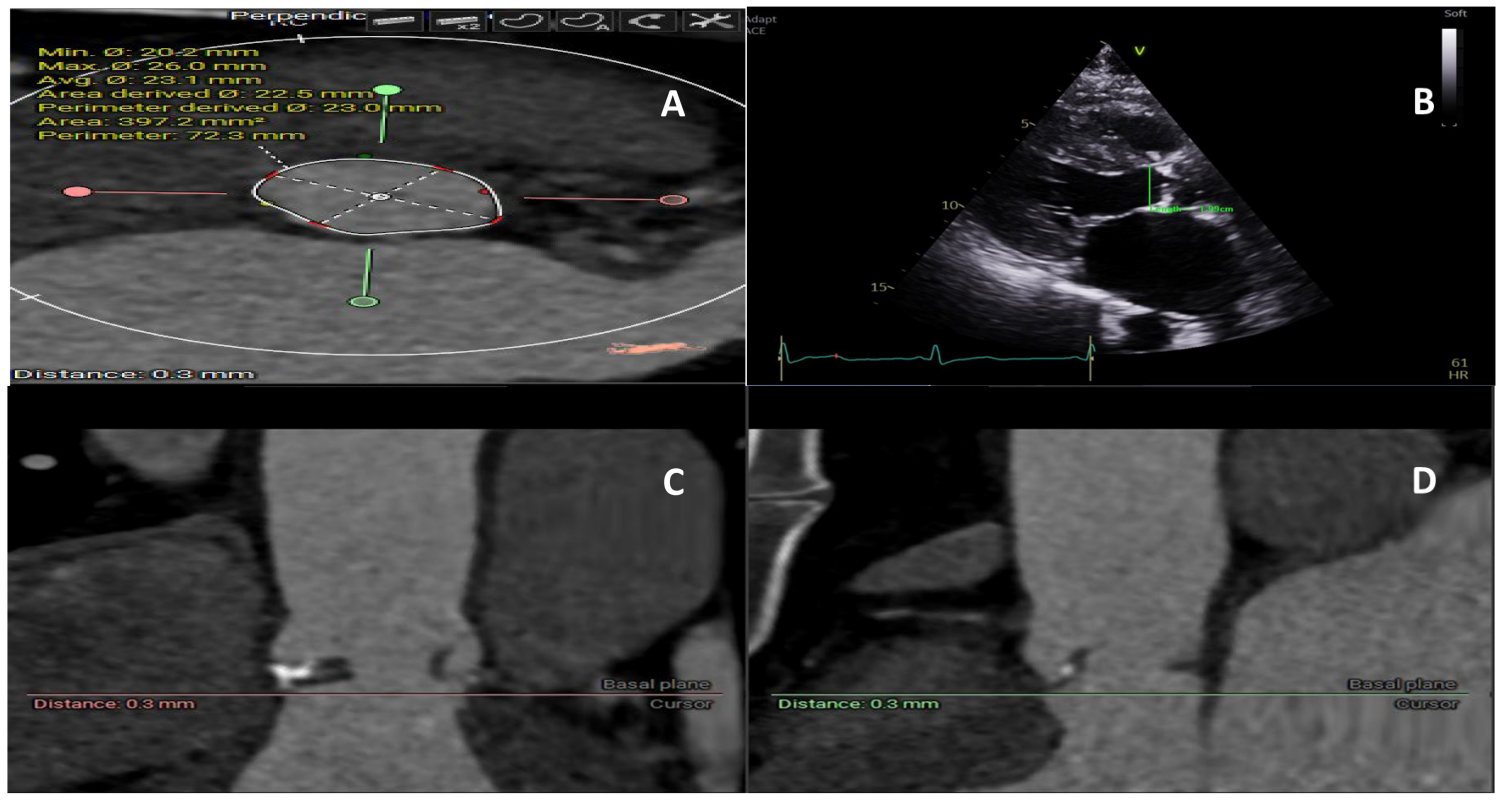
**Aortic annulus measurement using computed tomography and echocardiogram**. (A) Annulus size measurement on computed tomography providing area and perimeter. (B) Echocardiogram 2D measurement of the one axis of the annulus, (C) Computed tomography section provides a three-dimensional view of the annulus and its relationship to the surrounding structure. (D) Computed tomography section provides a three-dimensional view of the annulus and its relationship to the surrounding structure.

Despite recognizing the anatomical features of a small annulus more than five decades ago, there is currently no consensus on what defines a small annulus [5, 7]. These challenges stem from the discrepancy between the anatomical annulus used in surgical aortic valve replacement and imaging the annulus measured using echocardiography and computed tomography (CT). The junction, whereby the left ventricle meets the aortic root, represents the anatomical annulus and is used to attach the sewing ring of the surgical prosthesis. On the other hand, CT or echocardiography can be used to illustrate a virtual plane by connecting the lowest nadir of the aortic cusps. Historically, small aortic annulus was defined using echocardiography or direct sizing intraoperatively as less than 21–23 mm [7]. CT provides a three-dimensional structure of the aortic valve, and the aortic annulus area can be directly measured for valve sizing and procedural planning (Fig. 1). Subsequently, an area of less than 430 mm^2^ is being increasingly used to define small annulus in patients undergoing TAVI [11, 12]. For patients undergoing surgical aortic valve replacement (SAVR), CT is not routinely performed; however, recent data highlighted its potential role in improving valve sizing, reducing PPM, and decreasing interoperative variability [13].

Limited cohort studies exist that assess the true prevalence of small aortic annulus. The Simvastatin and Ezetimibe in Aortic Stenosis (SEAS) study included 1560 patients with predominately moderate aortic stenosis and evaluated the prognostic role of small aortic annulus [14], finding a prevalence for small aortic annulus of 17%. Importantly, a small aortic annulus was defined using the inner diameter of the aortic sinotubular junction indexed for body height, but resulted in an average aortic annulus diameter of 21 mm. On the other hand, contemporary data reported a small annulus in 40% of patients who underwent SAVR [15, 16]. This percentage is slightly lower in TAVI cohorts, and a small annulus was reported in one-third of patients [3, 9, 12]. This difference could be attributed to the imaging modalities used in surgical versus transcatheter heart valve studies [17]. By measuring the smallest diameter of the small annulus, echo-based methodology is more likely to underestimate the aortic annulus, particularly those with an elliptical shape (Fig. 1) [17].

It is well recognized that there are geographical variations in the prevalence of small aortic annulus: A direct comparison between European and Asian cohorts of patients undergoing TAVI highlighted a smaller aortic annulus and body surface area (BSA) in the Asian population [18]. Interestingly, this difference was also present between southern and northern European countries, although the BSA and gender distributions were comparable [19]. South European patients were almost seven times more likely to receive a small surgical prosthesis, but this did not translate into a difference in operative mortality [19].

No distinctive clinical features are associated with the small aortic annulus, except for female sex and low body surface area. Although diabetes, atrial fibrillation, renal failure, and high surgical profile risk were linked to small aortic annulus [7, 20], these characteristics were not evident in the Women’s International Transcatheter Aortic Valve Implantation (WIN-TAVI) registry or the Placement of Aortic Transcatheter Valves (PARTNER) study [21, 22]. The recently reported study on self-expanding versus balloon-expandable TAVR with small aortic annulus (SMART) had 87% of women in its cohort [11]. Real-world data highlighted comparable proportions of women in patients with small aortic annulus [12, 23]. The average BSA in patients with small aortic annulus was 1.8 m^2^ and is consistently lower than in patients with non-small aortic annulus [7, 11, 12].

## 3. The Hemodynamic Consequences of Small Aortic Annulus-PPM

**Fig. 2.**
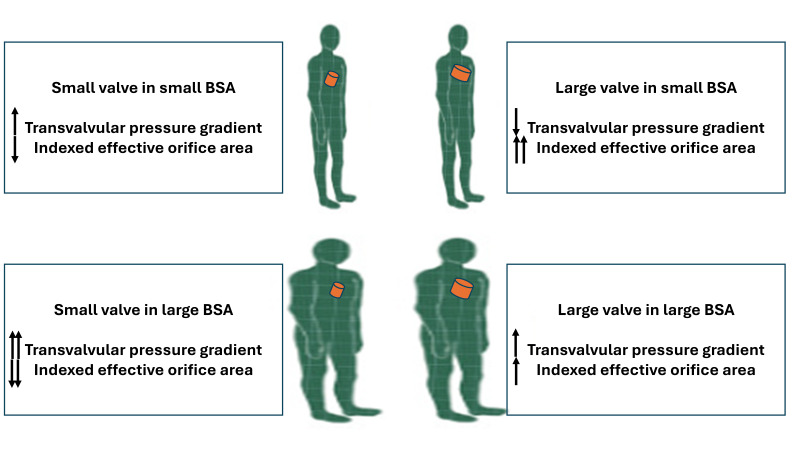
**Schematic illustration of consequent hemodynamic results in patients with various body surface areas and valve sizes**. The top row represents two patients with a small body surface area (BSA) who underwent aortic valve intervention with small (left) and large (right) prosthetic heart valves. The latter resulted in optimal hemodynamic status with low transvalvular pressure gradient (TPG) and large indexed effective orifice area (iEOA). The bottom row illustrates two patients with large BSAs who underwent aortic valve intervention with small (left) and large (right) prosthetic heart valves. The former patient had poor hemodynamic results with a very high TPG and very small iEOA.

Five decades ago, Rahimtoola [5] highlighted that almost all surgical prostheses have smaller *in vitro* EOAs than normal human valves. This is further compounded by *in vivo* tissue ingrowth and endothelization, resulting in the insertion of inherently stenotic prosthesis during cardiac surgery. This phenomenon may be tolerated in small or inactive individuals, but the hemodynamic consequences are suboptimal in large and active patients (Fig. 2) [5]. The concept of inserting a prosthetic valve with an EOA less than a normal human valve is called PPM [5, 6]. This definition considers body surface area; therefore, the relatively small prosthesis cannot meet the individual cardiac output at rest and exercise. PPM is currently defined as having an indexed EOA (iEOA) of less than 0.85 cm^2^ /m^2^[5, 6], a cutoff derived from moderate aortic stenosis in a native valve with a comparable iEOA [5, 6].

Importantly, PPM does not reflect anatomically or intrinsically defective prosthesis but rather a functional and hemodynamic abnormality leading to high gradient post-aortic valve intervention [5, 6]. Nevertheless, the relationship between the gradient and iEOA is not linear, and identifying patients with PPM should be primarily based on the iEOA since EOA influences the gradient, and the transvalvular flow can also determine gradient post-aortic valve intervention [6]. Transvalvular flow is linked to cardiac output, which is related to body surface area [6, 24]. Given their curvilinear relationship, there is no detected gradient following aortic valve intervention unless the EOA is reduced by 50% with an exponential increase in gradient once the EOA is less than 35% of the normal valve area [5]. This association is derived from Doppler echocardiography and not from invasive catheterization measurement since the latter is more prone to pressure recovery phenomenon. This, patients with similarly stenotic EOAs with different sizes of ascending aortas may have comparable transvalvular gradients on Doppler echocardiography, but a lower catheter gradient is likely to be present in those with small-size ascending aortas [25, 26].

During exercise, there is a disproportionate increase in gradient in patients with small iEOAs or PPM [6]. For example, a patient with an iEOA of 0.6 cm^2^/m^2^ may have a mean gradient of 80 mmHg during exercise compared to only 20 mmHg in an individual with an iEOA of 1.2 cm^2^/m^2^. Notably, the EOA of a bioprosthetic has the potential to increase during exercise [27]; however, this phenomenon becomes less evident in degenerative or mechanical valves [27]. Furthermore, follow-up studies suggested a reduction in cardiac index and worsening gradient in patients with PPM compared to those without PPM [6]. This occurred despite having a comparable cardiac index at baseline and a similar absolute reduction in EOAs over time [6].

PPM was recognized as an early adverse marker for left ventricle hypertrophy, and the reduction in left ventricle mass was linked to the iEOA post-surgery [28]. Notably, there was a differential response in left ventricle regression according to the iEOA, and patients with an iEOA greater than 0.8 cm^2^/m^2^ had a reduction in left ventricle mass index of more than 20% compared to merely 5% in those with an iEOA of less than 0.8 cm^2^ /m^2^ [28]. A reduction in left ventricle mass was evident as early as three months post-surgery [29]. This phenomenon may contribute to the additional 20% risk of re-admission with heart failure in patients with PPM [16, 30]. Moreover, this risk was even higher in patients with severe PPM (hazard ratio 1.59; 95% confidence interval 1.49–1.71; *p* < 0.001), and almost one-third of patients experienced hospitalization from heart failure over the 10-year follow-up [16, 30]. Additionally, the increased afterload in patients with PPM may have detrimental effects on left ventricle function [7, 31]. A similar stepwise increase in the risk of re-do aortic valve replacement (AVR) was evident in patients with moderate and severe PPM [16, 30]. The relative risk was reported to be up to three-fold in patients with severe PPM versus none [16, 30]. However, aortic valve reintervention was reported in only almost 3% of patients with severe PPM after the 10-year follow-up [16, 30]. Collectively, these adverse events translated into an increase in mortality, and large outcome studies reported a 30% increase in all-cause death at the 10-year follow-up [16, 30]. Notably, patients with severe PPM had a 35% survival rate at 10 years, which further decreased to less than 10% after 20 years [16, 30].

## 4. Procedural and Clinical Outcomes, Including Surgical Approach, in Patients with Small Aortic Annulus

Limited data exists that compares non-hemodynamic procedural outcomes of patients with small versus large aortic annulus. The SEAS study reported an increased risk of ischemic events, including cardiovascular death and non-hemorrhagic stroke, in patients with small aortic annulus [14]. Further, the authors proposed a possible link between small aortic annulus and subclinical atherosclerosis. However, this relationship was absent in subsequent studies reporting procedural and clinical outcomes according to annulus size [20, 21, 22]. Coronary artery disease, including previous percutaneous coronary intervention or coronary artery bypass grafts, peripheral vascular disease, and prior stroke, was at least numerically higher in patients with large aortic annulus [20, 21, 22].

The small aortic annulus was reportedly associated with an increased risk of coronary obstruction and annular rupture [7]. Importantly, this association was suggested, given that patients with coronary obstruction had small aortic annulus and more frequently received small prostheses [32, 33]. Nonetheless, small aortic annulus was not identified as an independent predictor of coronary obstruction [32, 33]. Similarly, other procedural adverse events, including stroke, bleeding, vascular complications, or conduction defects, were comparable in patients with small, intermediate, or large annuli [20, 21, 22].

Alternatively, moderate to severe paravalvular regurgitation was more consistently reported to be less common in patients with small aortic annulus [21, 22]. Data from Rogers *et al*. [34] highlighted no association between the rate of moderate to severe paravalvular regurgitation and annular size; however, their study had a relatively small sample size to challenge any definitive conclusions. The UK TAVI registry highlighted that large aortic annulus was an independent predictor of moderate to severe paravalvular regurgitation, and this relationship was linked to increased mortality [35]. Importantly, these data included old-generation transcatheter heart valves and the presence of a skirt, which reduced the incidence of paravalvular regurgitation and improved procedural outcomes [36]. Anatomically, it was suggested that patients with small aortic annulus were more likely to have complete apposition of the prosthesis against the aortic annulus, minimizing prosthesis–annulus incongruity and reducing paravalvular regurgitation [37, 38].

Over time, numerous surgical strategies have been used to manage patients with small aortic annulus. The earliest technique was aortic root enlargement (ARE), and different surgical techniques have been applied to ensure a larger prosthesis is implanted at the aortic annulus [39]. ARE has reduced the risk of PPM and increased iEOAs compared to patients without ARE [40, 41]. These hemodynamic effects were maintained at the 18-month follow-up, translating into significant regression in left ventricle mass [41]. Nonetheless, ARE did not reduce all-cause mortality or cardiovascular events at the 10-year follow-up [42, 43]. Moreover, a learning curve and prolonged surgical times are associated with ARE; nonetheless, it is considered a safe approach in modern AVR [7]. In a large study that included almost 2000 patients who underwent ARE, there was no association between procedural mortality and post-operative cardiovascular events between patients who underwent SAVR plus ARE and SAVR only [44]. ARE also has an impact on future reintervention, whereby it would enable larger implants of valve-in-valve TAVI and, thus, reduce the risk of PPM.

The alternative approach to minimize the risk of PPM was to redesign the surgical prosthesis by minimizing flow obstruction related to the presence of the surgical stent and sewing ring. Mounting the leaflets externally to the stent, such as microflow, crown, or trifecta, allowed for larger iEOAs and reduced risk of PPM [45]. While the three valves share a common design of supra-annular positioning to minimize stent and sewing ring interference with blood flow across the aortic valve, the single sheet of bovine pericardium resulted in a lower gradient in trifecta compared to the other two valves [45]. However, early degeneration was reported using trifecta with circumferential pannus formation, cusp tear, and leaflet calcification as its failure mechanisms [46].

Stentless valves were proposed as a treatment option for patients with small annulus. Indeed, by removing the stent, sewing the ring, and implanting the bioprosthetic leaflets using either the sub-coronary or full root technique, stentless valves may reduce the risk of PPM [47]. Compared with the supra-annular stented design, the stentless valve had a comparable mean gradient, and EOA early after surgery [48], and such hemodynamic performance was maintained at a one-year follow-up [49]. Additionally, left ventricle mass regression was similar between the two designs [49]. Nonetheless, stentless valves were associated with a high failure rate of more than 20% at a median follow-up of 11 years [50]. The survival rate was low at the 10-year follow-up at 46% despite the relatively young age at the time of SAVR [50, 51]. Moreover, redo surgery may require aortic root replacement, with the inevitable increase in mortality risk [7, 52].

The sutureless or rapid deployment valves are the most recent iteration in surgical bio-prosthesis. This design allowed for shorter surgical times, including cross-clamp and bypass time, while facilitating minimally invasive access. Previous studies reported good clinical and hemodynamic outcomes with sutureless valves, although the EOA was smaller than for those who underwent aortic root enlargement. Given its design, the risk of paravalvular regurgitation did not emerge, but consistently, there was an increased risk of conduction abnormalities, including permanent pacemakers in 20% of patients [53]. Moreover, the survival rate was acceptable. A meta-analysis of almost 3200 patients from seven observational studies highlighted a survival rate of 79.5% at 5 years follow-up. Although designed to reduce the risk of PPM, the sutureless valve was associated with a higher incidence of moderate to severe PPM compared to ARE [53]. Furthermore, a propensity score matching analysis demonstrated a higher residual gradient post-aortic valve intervention in patients who underwent sutureless valves versus TAVI [54]. This was associated with a more than five-fold increased risk of heart failure hospitalization at a 2-year follow-up, highlighting the hemodynamic advantage of using TAVI when managing aortic stenosis [54].

## 5. Transcatheter Versus Surgical Aortic Valve Intervention

**Fig. 3.**
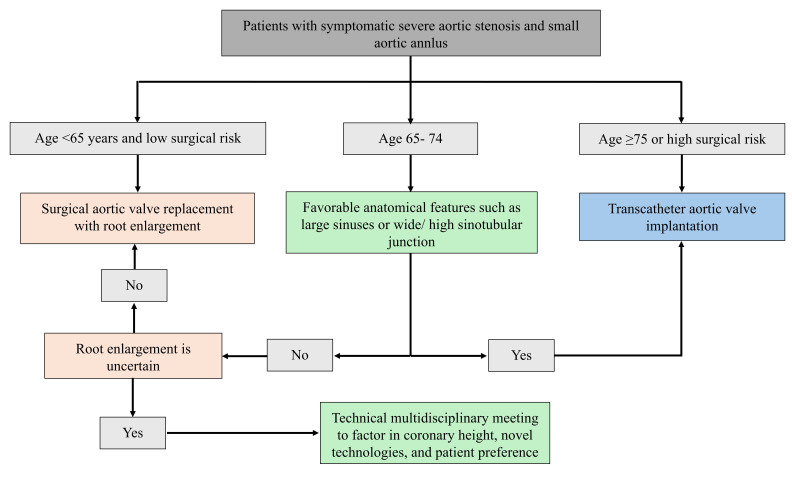
**Flowchart illustrating a treatment plan for patients with severe aortic stenosis and small aortic annulus undergoing aortic valve intervention**.

TAVI is currently considered the default treatment option for patients with severe aortic stenosis at intermediate-to-high surgical risk [1]. Recent data have also shown superior clinical outcomes associated with TAVI in patients with low surgical risk [55, 56]. Moreover, the hemodynamic performance in patients who underwent TAVI was better, with at least a three-fold increase in the risk of severe PPM in patients who underwent surgery (1.1% versus 3.5%; *p* = 0.008) [55]. Additionally, TAVI was associated with a significantly lower mean gradient and lower incidence of high residual transvalvular gradient, defined as a mean gradient ≥20 mmHg compared to SAVR [55]. These results were maintained at long-term follow-ups with lower mean gradients and larger EOAs in patients who underwent TAVI [57, 58]. Importantly, severe bioprosthetic valve dysfunction, defined as the composite of structural and non-structural valve deterioration, thrombosis, or endocarditis, was significantly lower in TAVI than in surgery [57, 58]. This was mainly driven by a higher incidence of severe PPM in surgical AVR compared to TAVI (31.9% vs. 10.2%; *p* < 0.01) [57, 58]. Mechanistically, the systematic oversizing in patients undergoing TAVI and the absence of a sewing ring would allow for larger EOAs and better hemodynamic results than surgical AVR. Fig. 3 illustrates a treatment plan for patients with severe aortic stenosis and small aortic annulus undergoing aortic valve intervention.

Given the superior hemodynamic results associated with first-generation transcatheter heart valves, recent data reinforced its advantage when using second-generation devices. O’Hair *et al*. [8] reported the incidence of structural valve deterioration (SVD) from a large study of almost 4500 patients with available echocardiogram data from a 5-year follow-up. SVD was significantly lower in patients who underwent TAVI compared to SAVR (2.20% vs. 3.38%, hazard ratio (HR) 0.46, 95% CI (0.27–0.78); *p* = 0.004) [8]. Such differences were more pronounced in patients with a small annulus (1.32% vs. 5.84%, HR 0.21, 95% CI (0.06–0.73); *p* = 0.02) [8]. More importantly, the presence of SVD was associated with double the risk of all-cause mortality, more than a two-fold increased risk of heart failure hospitalization, and an 80% increase in cardiovascular mortality [8].

Recently, the Transcatheter Aortic Valve Replacement Versus Surgical Aortic Valve Replacement for Treating Elderly Patients with Severe Aortic Stenosis and Small Aortic Annuli (VIVA) trial investigated the clinical and hemodynamic outcomes of using either treatment in patients with small annulus [59]. The study was the first randomized trial to address this question and included 151 patients with severe aortic stenosis and a mean aortic annulus diameter of <23 mm [59]. The incidence of severe PPM was numerically higher (almost doubled) in the SAVR compared to the TAVI group (10.3% vs. 5.6%); nonetheless, this did not reach statistical significance (*p* = 0.30) (See Table 1 (Ref. [11, 48, 49, 59, 60, 61, 62, 63, 64]) summarizing landmark studies assessing the role of various strategies in aortic valve intervention) [59]. Similarly, there was no difference in mortality rate, stroke, or cardiac hospitalization over a median of 2 years of follow-up [59]. As highlighted by the authors, the study had a small sample size, and its results need to be interpreted within this context. Unsurprisingly, 93% of the included patients were women, underlining the strong association between small annulus and women. The Randomized researcH in womEn all comers wIth Aortic stenosis (RHEIA) trial evaluated the clinical outcomes of aortic valve intervention using TAVI or surgery in women [65]. The results were recently presented at a European Society of Cardiology meeting and demonstrated superior outcomes from using TAVI in reducing the composite endpoint of all-cause mortality, stroke, heart failure, or valve-related rehospitalization after one year. The difference was mainly driven by rehospitalization for heart failure or valve-related symptoms, with no difference in the incidence of death or stroke between the two treatment arms. However, long-term follow-up is needed to assess the hemodynamic results of TAVI and surgery in this group.

**Table 1.** **Landmark surgical and transcatheter studies that assessed the role of aortic valve platform in patients undergoing aortic valve intervention**.

Study/year	Comparative groups	Age	Rationale/primary endpoint	Follow up	Outcome
Narang *et al*./2008 [63]	62 pts: Group A (n = 30) used the Freestyle porcine stentless tissue valve (Medtronic, Inc); Group B (n = 32) used the Carpentier–Edwards Perimount Standard stented aortic valve.	Group A, 55 ± 8; Group B, 56 ± 10	Compared stentless and stented bioprostheses, clinical outcomes, hemodynamic performance, and postoperative left ventricular mass regression.	18 ± 3 months	For patients with left ventricular impairment or a small aortic annulus, stentless bioprostheses might allow for more significant improvement in left ventricular function postoperatively.
Suri *et al*./2012 [64]	300 pts: Edwards Magna (n = 100), Sorin Mitroflow (n = 101), St. Jude Epic (n = 99).	76 ± 8 years	Early postoperative hemodynamic differences among current third-generation porcine and pericardial aortic valve prostheses.	NA	The three valves studied performed equally well in patients with a small (21 mm) aortic annulus. The Magna valve had a slightly lower mean gradient in those with larger annular size (>23 mm).
Tasca *et al*./2014 [48]	40 pts with a native aortic annulus diameter ≤2.3 cm were randomized to receive St. Jude Medical Trifecta stented prosthesis (n = 20) or a Medtronic Freestyle stentless prosthesis (n = 20).	81 ± 4 years	Compared the hemodynamic performance of the Trifecta valve with that of the Freestyle valve in patients with an aortic annulus ≤2.3 cm.	NA	The Trifecta valve showed slightly better hemodynamics and larger indexed effective orifice areas (iEOAs); patient prosthesis mismatch (PPM) occurred in two patients in the Freestyle group and three in the Trifecta group.
Tasca *et al*./2015 [49]	40 pts with a native aortic annulus diameter ≤2.3 cm were randomized to receive St. Jude Medical Trifecta stented prosthesis (n = 20) or a Medtronic Freestyle stentless prosthesis (n = 20).	81 ± 4 years	Compared the hemodynamic performance of Trifecta vs. Freestyle valves at one year in patients with an aortic annulus ≤2.3 cm.	1 year	No differences between Trifecta and Freestyle were found in one-year hemodynamics and indexed effective orifice areas (iEOAs). Only moderate patient prosthesis mismatch was noted, which affected 3 patients in each group.
CHOICE Randomised Clinical Trial [60]/2020	241 pts: Edwards Sapien XT valve BEV (n = 121) vs. Medtronic CoreValve SEV (n = 120). The CHOICE-Extend registry [62] (Evolut R [ER], n = 100; SAPIEN 3 [S3], n = 334).	81.5 ± 6.2 years	Compared the performance of a balloon-expandable (BE) transcatheter heart valve (THV) versus a self-expanding (SE) THV. The primary endpoint was device success, with assessment of clinical outcomes and echocardiographic evaluation of valve function and THV durability.	5 years	The trial revealed clinical outcomes after TAVR with early-generation BE and SE valves that were not statistically significantly different, with limited statistical power.
					Flow hemodynamics were significantly better with the SE valve.
					Moderate or severe structural valve deterioration was uncommon but occurred more frequently with the BE valves.
					On multivariate analysis, transcatheter aortic valve replacement with the ER in small annuli was associated with a lower rate of patient prosthesis mismatch than with the S3, with no increased risk for PVR.
SOLVE-TAVI trial/2020 [61]	447 pts: 225 pts were randomly assigned to SEV and 222 pts to BEV implantation.	81 ± 5 years	Comparison between newer generation self-expandable Evolut R vs. balloon-expandable valves Sapien 3 in TAVI.	60 months	At 30 days, the primary endpoint of all-cause mortality, stroke, moderate or severe PVL, and permanent pacemaker implantation was equivalent between SEV and BEV (28.4% vs. 25.9%; rate difference –2.51 (90% confidence interval (CI) –9.65 to 4.53); *p*-equivalence = 0.04).
					New generation SEV, in comparison to BEV, are equivalent from the endpoint view.
VIVA trial/2024 [59]	151 pts with severe aortic stenosis and SAA (mean diameter <23 mm), TAVR (n = 77) versus surgical aortic valve replacement (SAVR) (n = 74).	75.5 ± 5.1 years	Compared the hemodynamic and clinical outcomes between transcatheter aortic valve replacement (TAVR) and SAVR in patients with a SAA. Clinical events were presented as the secondary outcomes.	2 years	1. There were no differences between groups in the rate of severe prosthesis patient mismatch (TAVR, 4 (5.6%); SAVR, 7 (10.3%); *p* = 0.30).
					2. No moderate–severe aortic regurgitation was found in either group.
					3. No differences were found in mortality rate (TAVR, 1 (1.3%); SAVR, 1 (1.4%); *p* = 1.00) and stroke (TAVR, 0; SAVR, 2 (2.7%); *p* = 0.24) at 30 days.
					4. After a median follow-up of 2 years, there were no differences in mortality rate (TAVR, 7 (9.1%); SAVR, 6 (8.1%); *p* = 0.89), stroke (TAVR, 3 (3.9%); SAVR, 3 (4.1%); *p* = 0.95), and cardiac hospitalization (TAVR, 15 (19.5%); SAVR, 15 (20.3%); *p* = 0.80).
					5. There were no significant differences between groups in the rate of severe PPM or moderate–severe AR (TAVR, 4 (5.6%); SAVR, 7 (10.3%); mean difference, –4.74 (95% CI, –13.69 to 4.21); *p* = 0.30).
SMART trial/2024 [11]	716 pts: self-expanding supra-annular valve or a balloon-expandable valve in patients with an aortic valve annulus area of 430 mm^2^ or less in a 1:1 ratio.	80 years	The co-primary endpoints were a composite of death, disabling stroke, or rehospitalization for heart failure (tested for noninferiority) and a composite endpoint measuring bioprosthetic valve dysfunction (tested for superiority).	12 months	The self-expanding supra-annular valve was non-inferior to a balloon-expandable valve concerning clinical outcomes, but it was superior concerning bioprosthetic valve dysfunction.

Abbreviations: pts, patients; NA, no applicable; TAVR, transcatheter aortic valve replacement; PVR, paravalvular regurgitation; BEV, balloon-expandable valve; SEV, self-expendable valve; TAVI, transcatheter aortic valve implantation; PVL, paravalvular leak; SAA, small annular area; PPM, patient prosthetic mismatch.

## 6. Balloon-Expandable Versus Self-Expanding Valves for Small Annulus

TAVI emerged as a dominant treatment for severe aortic stenosis given its superior clinical and hemodynamic results; nonetheless, the choice of TAVI platform remains a controversial topic in relation to patients with small annulus. Although comparatively lower than for surgery, moderate or severe PPM incidence could be detected in up to 35% of patients undergoing TAVI [11]. Recent data from the myocardial recovery following transcatheter aortic valve replacement for severe aortic stenosis (RECOVERY-TAVR) study highlighted the clinical and echocardiographic features of patients with severe PPM following TAVI [66]. In this registry of 963 TAVI patients (almost 50% had balloon-expandable valve, BEV), the incidence of severe PPM was 7.7%, with no difference in the clinical characteristics between patients who developed or did not develop PPM at baseline [66]. Patients with severe PPM were more likely to receive BEV and had low stroke volume and low transaortic flow rate [66]. The latter was recently shown to be an independent predictor of all-cause mortality in patients undergoing TAVI [24]. Small prosthesis, low-indexed stroke volume, and small left ventricle outflow tract were all independent predictors of PPM in this study [24]. This highlights the detrimental effect of small anatomy and/or small transcatheter heart valves on hemodynamic post-TAVI.

Few registries investigated the short and long-term outcomes of BEV versus self-expanding valves (SEV) with inconsistent results [23, 67]. The Comparison of Transcatheter Heart Valves in High-Risk Patients with Severe Aortic Stenosis: Medtronic CoreValve vs. Edwards SAPIEN XT (CHOICE) trial randomized 241 high-risk patients and compared old generation BEV versus SEV [60, 68]. Although device success was higher in patients undergoing BEV, the 5-year outcomes were comparable between the two platforms. The hemodynamic performance was better in patients undergoing SEV and had a lower incidence of clinical valve thrombosis and severe structural valve degeneration [60]. Similar results were reported with second-generation TAVI from the compariSon of secOnd-generation seLf-expandable vs. balloon-expandable Valves and gEneral vs. local anaesthesia in Transcatheter Aortic Valve Implantation (SOLVE-TAVI) trial [61]. Importantly, the CHOICE and CHOICE-Extend highlighted that using SEV compared to BEV was independently associated with a 53% and 84% reduction in PPM in large and small annuli, respectively [62].

The SMART trial addressed whether the TAVI platform impacts clinical and hemodynamic outcomes using the latest generation SEV compared to BEV in patients with symptomatic severe aortic stenosis and a small aortic valve annulus [11]. A total of 716 patients, 87% women, with an aortic annulus area of ≤430 mm^2^, were randomized to receive either SEV or BEV. The primary clinical endpoint was not different between the two groups, and the incidence of death, disabling stroke, or rehospitalization for heart failure at 12 months was 9.4% in SEV compared to 10.6% in BEV [11]. On the other hand, the co-primary hemodynamic endpoint of bioprosthetic valve dysfunction was significantly lower in SEV compared to BEV (9.4% vs. 41.6%; *p* < 0.001 for superiority) [11]. Valve performance was also better in SEV, defined as a lower mean gradient, larger EOA, a lower percentage of hemodynamic structural valve dysfunction, and moderate-to-severe PPM [11]. While the hemodynamic superiority was not translated into a difference in clinical outcomes, it is plausible that longer follow-ups may illustrate the impact of severe PPM on mortality and re-admission from heart failure. A recent Transcatheter Valve Therapy (TVT) Registry analysis highlighted the association between severe PPM and mortality as early as three years [69].

Furthermore, the SMART trial showed significantly lower total aortic regurgitation in patients who underwent SEV. Mild or greater aortic regurgitation at 12 months was evident at 14.1% compared to 20.3% in BEV. The risk of pacemakers was numerically higher in patients undergoing SEV versus BEV (12.1% vs. 7.8%) platform and was consistent across major studies [11]. Although the quality of life was comparable, the proportion of patients with improved scores at 12 months was significantly higher in SEV versus BEV [11]. Recent real-world data provided mechanistic insights into the enhanced functional status associated with SEV compared to BEV [12]. Omari *et al*. [12] reported larger cardiac output and cardiac index in patients with small annulus who underwent TAVI with SEV compared to BEV. This difference was more evident in patients with a large BSA and underlies the importance of tailoring treatment in patients with small aortic annulus [12].

## 7. Conclusion

Patients with severe aortic stenosis and a small aortic annulus are a challenging anatomic subset that requires careful evaluation and planning. Treatment options include both surgical and transcatheter valve implantation. The decision on treatment modality should consider the hemodynamic consequences of any aortic valve intervention to improve the symptoms of patients, ensure the longevity of the bioprosthetic valve, and potentially reduce long-term clinical outcomes.
